# A Novel Effective and Safe Vaccine for Prevention of Marek’s Disease Caused by Infection with a Very Virulent Plus (vv+) Marek’s Disease Virus

**DOI:** 10.3390/vaccines9020159

**Published:** 2021-02-16

**Authors:** Yifei Liao, Sanjay M. Reddy, Owais A. Khan, Aijun Sun, Blanca Lupiani

**Affiliations:** 1 Department of Veterinary Pathobiology, College of Veterinary Medicine & Biomedical Sciences, Texas A&M University, College Station, TX 77843, USA; liao.yifei@tamu.edu (Y.L.); SReddy@cvm.tamu.edu (S.M.R.); owais.khan@tvmdl.tamu.edu (O.A.K.); 2 Texas A&M Veterinary Medical Diagnostic Laboratory, Canyon, TX 79016, USA; 3 College of Animal Sciences and Veterinary Medicine, Henan Agricultural University, Zhengzhou 450002, China

**Keywords:** Marek’s disease virus, Meq, vIL8, lymphoid organ atrophy, vaccine

## Abstract

Marek’s disease virus (MDV) is a highly contagious alphaherpesvirus that causes rapid onset lymphoma in chickens. Marek’s disease (MD) is effectively controlled using vaccination; however, MDV continues to break through vaccinal immunity, due to the emergence of highly virulent field strains. Earlier studies revealed that deletion of the *meq* gene from MDV resulted in an attenuated virus that protects against MD in chickens challenged with highly virulent field strains. However, the *meq* deleted virus retains the ability to induce significant lymphoid organ atrophy. In a different study, we found that the deletion of the *vIL8* gene resulted in the loss of lymphoid organ atrophy in inoculated chickens. Here, we describe the generation of a recombinant MDV from which both *meq* and *vIL8* genes were deleted. In vitro studies revealed that the *meq* and *vIL8* double deletion virus replicated at levels similar to the parental very virulent plus (vv+) virus. In addition, in vivo studies showed that the double deletion mutant virus (686BAC-ΔMeqΔvIL8) conferred protection comparable to CVI988, a commercial vaccine strain, when challenged with a vv+ MDV virus, and significantly reduced lymphoid organ atrophy, when compared to *meq* null virus, in chickens. In conclusion, our study describes the development of a safe and effective vaccine candidate for prevention of MD in chickens.

## 1. Introduction

Marek’s disease (MD) is caused by the highly contagious Marek’s disease virus (MDV) and is characterized by immunosuppression, neurological disease, and rapid onset of T cell lymphoma in visceral organs and skin [1,2,3]. MDV is an alphaherpesvirus and is classified in the *Mardivirus* genus of the *Herpesviridae* family. There are three highly related species within the *Mardivirus* genus: MDV type 1 (MDV-1, also known as *Gallid alphaherpesvirus* 2, GaHV-2) includes pathogenic strains and their derivatives; MDV type 2 (MDV-2 or GaHV-3) consists of non-oncogenic viruses of chickens; and turkey herpesvirus (HVT, also known as *Meleagrid alphaherpesvirus* 1, MeHV-1) [4]. Oncogenic MDVs are further classified into four pathotypes depending on their virulence in vaccinated chickens: mildly virulent (m), virulent (v), very virulent (vv), and very virulent plus (vv+) [5]. 

MD is the first tumor disease that has been successfully controlled using vaccination [6], and strains from MDV-1 (refers to MDV in following text), MDV-2, and HVT have been used as vaccines to protect against MD [3,7,8]. An MDV cell culture passage attenuated viral strain, HPRS-16/att, was first used to protect chickens against challenge with v MDV strains [9]. HVT started being used in the 1970s and was followed by the HVT/MDV-2 bivalent vaccine in the 1980s to protect against emerging vv MDV field isolates [10,11,12]. In the early 1990s, emergence of vv+ field strains resulted in the introduction, in the United States, of CVI988/Rispens, a cell culture passage attenuated MDV virus and the gold standard among MD vaccines [13,14]. More recently, to increase the replication and vaccine efficacy of CVI988, a novel chimeric vaccine, CVRM (Prevexxion RN, Boehringer Ingelheim), was generated by inserting the reticuloendotheliosis (REV) long terminal repeat (LTR) into the CVI988 genome and is currently commercially available [15]. Although currently available vaccines are able to keep MD under control, MDV continues to evolve and there is a need to develop improved next generation vaccines.

Advances in molecular cloning technology have allowed the manipulation of the MDV genome and deletion of MDV-specific gene or genes involved in pathogenesis. The genomic structure of MDV consists of unique long (U_L_) and unique short (U_S_) regions, each flanked by inverted repeat regions (terminal repeat long (TR_L_), internal repeat long (IR_L_), internal repeat short (IR_S_), and terminal repeat short (TR_S_) regions, respectively) [16,17]. MDV repeat long regions, TR_L_ and IR_L_ encode a number of unique genes like *meq* (MDV EcoRI Q), *pp38* (phosphorylated protein 38 kDa), *vIL8* (virus-encoded interleukin 8), and *vTR* (virus-encoded telomerase RNA subunit), which have been shown to play important roles in MDV pathogenesis [18,19,20,21]. 

MDV *meq* encodes a 339 amino acid protein which consists of a DNA binding domain, nuclear and nucleolar localization signal sequences, a basic leucine zipper (bZIP) domain, and a C-terminal proline-rich domain [22]. The bZIP domain interacts with itself or the bZIP domain of c-Jun or c-Fos proteins to form homodimers and heterodimers, respectively, regulating the transcription of target genes [23,24,25]. In addition, both homo- and heterodimers of Meq have been shown to play a role in the transformation of chicken lymphocytes [23,24,25]. Overexpression of Meq also resulted in the transformation of rodent (Rat-2 and NIH/3T3 cells) and chicken (DF-1 cells) fibroblast cell lines in vitro [26,27,28]. The direct role of Meq in MDV transformation was demonstrated by the generation of a *meq* deletion mutant virus, rMd5∆Meq, which completely lost its ability to induce tumors in highly susceptible MDV maternal antibody negative (Ab–) chickens [21]. Interestingly, rMd5∆Meq provided protection superior to CVI988 in chickens challenged with the vv+ MDV 648A strain under experimental and field conditions [29,30]. Although rMd5∆Meq is an effective vaccine, it retains the ability to establish a robust early cytolytic infection resulting in lymphoid organ (bursa and thymus) atrophy in Ab– chickens [31,32]. This property raises safety concerns and limits the commercialization and licensing of this vaccine candidate. Attempts to use serial cell culture passage, to eliminate rMd5∆Meq-induced lymphoid organ atrophy, resulted in an attenuated virus, but also in a reduction of protective efficacy [32]. To improve the safety of rMd5∆Meq as a vaccine, other molecular factors need to be considered.

Viral interleukin-8 (vIL8), a homology of cellular IL8, is an important MDV gene involved in MDV-induced lymphomagenesis. *vIL8* is located in the repeat long regions of the MDV genome and consists of three exons separated by two introns [20]. Deletion of MDV *vIL8* in Md5, a vv MDV, showed that vIL8 is important for the establishment of early cytolytic infection, and the resulting mutant virus did not cause lymphoid organ atrophy and provided significant protection against vv MDV challenge [33,34]. However, vIL8 null mutant MDV retains the ability to cause tumors in highly susceptible chickens [20,33], and thus is not suitable as a vaccine. On the basis of the hypothesis that vIL8 contributes to the lymphoid organ atrophy caused by parental and MDV-∆Meq mutant viruses, we constructed a *meq* and *vIL8* double deletion MDV (686BAC-∆Meq∆vIL8) using the bacterial artificial chromosome (BAC) clone of 686, a vv+ MDV strain [35]. Our results show that 686BAC-∆Meq∆vIL8 was fully attenuated, with regards to both tumors and lymphoid organ atrophy, and conferred protection comparable to CVI988/Rispens against a vv+ MDV challenge. This strategy provides evidence for the development of safe and efficacious vaccines against vv+ MDV strains.

## 2. Material and Methods

### 2.1. Cells and Viruses

Primary chicken embryonic fibroblasts (CEF), prepared from 10-day-old chicken embryos, were used for BAC DNA transfection, virus propagation, and virus reactivation assay. Recombinant viruses 686BAC-ΔMeq, 686BAC-ΔvIL8, and 686BAC-ΔMeqΔvIL8 were generated from 686BAC, the BAC clone of a vv+ MDV strain [35]. MDV 686 strain, used as challenge virus, and CVI988/Rispens vaccine virus were obtained from the Avian Disease and Oncology Laboratory, United States Department of Agriculture (East Lansing, MI, USA). CEF were cultured at 37°C in Leibowitz-McCoy (LM) medium supplemented with penicillin-streptomycin and 5% bovine calf serum. 

### 2.2. Construction of meq and/or vIL8 Single and Double Deletion Viruses

To delete *meq* and *vIL8* genes, individually or together, from the 686BAC, a two-step Red-mediated recombination procedure was performed, as previously described [36,37]. Briefly, the *Kan^R^*-*I-SecI* cassette was amplified from pEPkan-S and electroporated into the 686BAC-containing *E. coli*, where *meq* or *vIL8* genes were replaced with the *Kan^R^*-*I-SecI* cassette. The *Kan^R^* sequence was then deleted by the addition of arabinose to generate 686BAC with a single copy of *meq* or *vIL8* genes. Due to the presence of two copies of *meq* and *vIL8* in the MDV genome, this procedure was repeated to generate 686BAC-∆Meq and 686BAC-∆vIL8 mutants where both copies of *meq* or *vIL8* were deleted. Then, 686BAC-∆Meq was used as the backbone to generate the *meq* and *vIL8* double deletion mutant (686BAC-ΔMeqΔvIL8) using the same procedure. All mutant BAC clones were screened by PCR, followed by DNA sequencing and restriction fragment length polymorphism (RFLP) to confirm the deletion of *meq* and *vIL8* and the absence of unexpected mutations. Primers used to construct all mutant BAC clones are shown in Table 1. All BAC DNAs were transfected into CEF to produce *meq* or *vIL8* single and double deletion mutant viruses.

### 2.3. Immunofluorescence (IFA) and Immunohistochemistry (IHC) Assays

IFA was carried out as previously described with modifications [38]. Briefly, infected or transfected CEF were washed with phosphate-buffered saline (PBS) and fixed with ice-cold acetone:methanol (6:4) for 10 min. After blocking with non-fat dry milk, the cells were incubated with MDV pp38 monoclonal antibody (1:500) for 1 hour, followed by another hour of incubation with goat anti-mouse fluorescein isothiocyanate (FITC)-labeled secondary antibodies (KPL, Gaithersburg, MD, USA). Cells were then washed three times with PBS and examined under a fluorescence microscope. 

For IHC, lymphoid organs (thymus, spleen, and bursa of Fabricius) and skin with feather follicles were embedded in optimal cutting temperature compound (Tissue-Tek, OCT, Sakura Finetek, Torrance, CA, USA), frozen immediately in liquid nitrogen, and stored at −80 °C until use. Six to eight µm-thick cryostat sections were generated and subjected to immunostaining with MDV pp38 monoclonal antibody (1:3200) and the Vectastain ABC kit (Vector Laboratories, Burlingame, CA, USA) according to the manufacturer’s instructions. 

### 2.4. In Vitro Growth Kinetics

In vitro growth kinetics of 686BAC, 686BAC-∆Meq, 686BAC-∆vIL8, and 686BAC-∆Meq∆vIL8 viruses were determined as described previously [21]. Briefly, CEF, seeded on 60 mm plates, were inoculated with 100 plaque-forming units (PFU) of the different viruses. On days 1, 2, 3, 4, and 5 post infection, infected cells were trypsinized, followed by serial dilutions and co-culture with fresh CEF, and plaques were counted 7 days post infection.

### 2.5. MDV Genome Copy Numbers

To compare the replication of 686BAC, 686BAC-∆Meq, 686BAC-∆vIL8, and 686BAC-∆Meq∆vIL8 viruses in vivo, one-day-old specific pathogen free (SPF) and MDV maternal antibodies free (Ab–) chickens (Charles Rivers Laboratories, Wilmington, MA, USA) were inoculated subcutaneously with 2000 PFU of each virus; one group was not inoculated and served as the negative control. Three chickens from each group were euthanatized at 5, 14, or 56 days post-inoculation (dpi) and spleen samples were collected. Genomic DNA was extracted from chicken splenocytes using the phenol-chloroform method, and MDV genome copy number was measured by quantitative polymerase chain reaction (qPCR) using primers specific for MDV-infected cell protein 4 (*ICP4*) and chicken *GAPDH* genes (Table 1) [39,40]. All qPCR assays were carried out in the Bio-Rad iCycler iQ Multicolor Real-Time Detection System (Bio-Rad, USA), using iTag SYBR supermix buffer (Bio-Rad, USA). Results are presented as the ratio of MDV DNA copy numbers divided by the copy number of GAPDH, with error bars representing standard error of the mean (SEM).

### 2.6. Virus Reactivation Assay

To examine the reactivation efficacy of mutant viruses, 3 chickens from each experimental group were randomly selected and bled at 14 dpi for virus reactivation assay. Briefly. CEF monolayers (seeded on 35 mm plates, in duplicate) were co-seeded with 10^6^ peripheral blood lymphocytes (PBL) isolated from 686BAC, 686BAC-ΔMeq, 686BAC-ΔvIL8, or 686BAC-ΔMeqΔvIL8 viruses inoculated chickens (SPF, Ab–) or negative control chickens at 14 dpi. Plaques were counted 7 days after infection, and data were presented as average plaque numbers ± SEM.

### 2.7. Pathogenesis of meq or vIL8 Single and Double Deletion Mutant Viruses in SPF Ab– Chickens

One-day-old SPF Ab– chickens were wing-banded and randomly sorted into 5 experimental groups. Chickens were inoculated subcutaneously with 2000 PFU of parental 686BAC, 686BAC-∆Meq, 686BAC-∆vIL8, and 686BAC-∆Meq∆vIL8 viruses, or were not inoculated and kept as negative control. All animal experiments were carried out in accordance with Texas A&M University Institutional Animal Care and Use Committee (IACUC) approved protocol.

*a*  *Lymphoid organ atrophy*: To evaluate the effect of virus replication in lymphoid organ atrophy, 5 randomly selected chickens from each experimental group were euthanatized at 14 dpi, and thymus and bursa were weighed. Results were presented as the average ratio of lymphoid organs weight to body weight of 5 chickens multiplied by one hundred.
*b*  *Pathogenesis*: To compare the pathogenic properties of parental 686BAC, 686BAC-∆Meq, 686BAC-∆vIL8, and 686BAC-∆Meq∆vIL8 viruses, the mortality of each experimental group was recorded daily for 65 days. All chickens that died during the experiment or were euthanized at the end of the experiment were necropsied and examined for MD associated gross tumors in visceral organs and nerves.

### 2.8. Vaccine Protection Experiments

To study the protection efficacy of 686BAC-∆Meq∆vIL8, one-day-old SPF Ab– and MDV maternal-antibody-positive (Ab+) commercial White Leghorn male chickens (Hyline International, Bryan, TX, USA) were not vaccinated or vaccinated with 2000 PFU of 686BAC-∆Meq, 686BAC-∆Meq∆vIL8, or CVI988 by the subcutaneous route. Five days later, vaccinated and unvaccinated chickens were challenged subcutaneously with 500 PFU of 686. Chickens that died or survived to the end of the experiment (56 days post challenge) were necropsied and examined for MD associated gross tumors. Vaccine protection efficacy was expressed as protective index (PI), as previously described [29,32]. 

### 2.9. Data and Statistical Analysis

For in vitro growth kinetics, data represent an average of duplicates and were analyzed by ANOVA for each individual time point. The relative lymphoid organs to body weight ratios were averaged over 5 independently collected samples in each group and analyzed by Student *t* test. The trends of the chicken survival curve were examined with LogRank and Wilcoxon tests. The protective index (PI) among the different groups was analyzed by Chi Square test. All statistical analyses were performed with GraphPad Prism software (GraphPad Software, Inc. La Jolla, CA, USA). A value of *p* < 0.05 was considered statistically significant. 

## 3. Results

### 3.1. Construction and In Vitro Characterization of meq or vIL8 Single and Double Deletion Viruses

**Figure 1:**
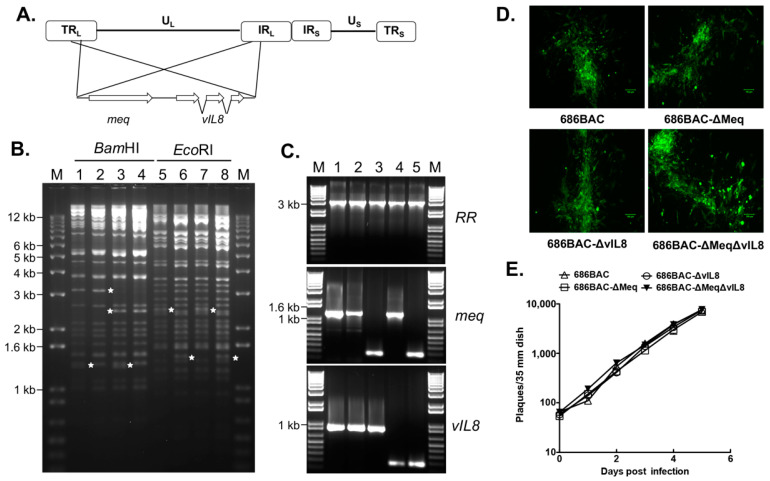
In vitro characterization of *meq* and *vIL8* single and double deletion mutant viruses. (**A**) Genomic structure of serotype 1 Marek’s disease virus (MDV) and location of *meq* and *vIL8* genes. U_L_: unique long; U_S_: unique short; TR_L_ and IR_L_: terminal and internal repeat long; TR_S_ and IR_S_: terminal and internal repeat short. (**B**) Genomic analysis of *meq* and/or *vIL8* deletion viruses. DNA of 686BAC (lanes 1 and 5), 686BAC-ΔMeq (lanes 2 and 6), 686BAC-ΔvIL8 (lanes 3 and 7) and 686BAC-ΔMeqΔvIL8 (lanes 4 and 8) were digested with *Bam*HI or *Eco*RI, followed by agarose gel electrophoresis. Asterisks indicate different bands due to deletion of the *meq* and/or *vIL8* genes. M: 1 kb plus ladder. (**C**) PCR analysis of viral genome isolated from infected chicken embryonic fibroblasts (CEF), using primers specific for MDV ribonucleotide reductase (*RR*), and *meq* and *vIL8* flanking primers. Lane 1: 686 virus; lane 2: 686BAC virus; lane 3: 686BAC-ΔMeq virus; lane 4: 686BAC-ΔvIL8 virus; lane 5: 686BAC-ΔMeqΔvIL8 virus. (**D**) Immunofluorescence assay (IFA). 686BAC, 686BAC-ΔMeq, 686BAC-ΔvIL8 or 686BAC-∆Meq∆vIL8 BAC transfected CEF were subjected to IFA using MDV pp38 specific monoclonal antibody and FITC conjugated secondary antibody. Scale bar = 100 µm. (**E**) *In vitro* growth kinetics. CEF were infected with 100 plaque-forming units (PFU) of 686BAC, 686BAC-ΔMeq, 686BAC-ΔvIL8 and 686BAC-ΔMeqΔvIL8 viruses. Infected cells were trypsinized, diluted, and co-seeded with fresh CEF at the indicated time points, and plaques were counted 6 days post infection. Each point represents two independent experiments and data present average plaque numbers ± standard error of the mean (SEM).

MDV *meq* and *vIL8* genes (Figure 1A) alone or in combination were deleted from 686BAC DNA via a two-step Red-mediated recombination method to generate 686BAC-∆Meq, 686BAC-∆vIL8, and 686BAC-∆Meq∆vIL8, respectively. To confirm the absence of an unexpected recombination associated with the deletion processes, BAC DNAs were subjected to RFLP analysis using *Bam*HI and *Eco*RI enzymes. RFLP results showed that the patterns obtained correspond exactly to those predicted by in silico analysis without unexpected rearrangements in the mutant MDV genomes (Figure 1B). The *Bam*HI fragment corresponding to the *meq* gene in 686BAC is 5133 bp and 1288 bp, whereas in 686BAC-∆Meq, we expect a single 5404 bp fragment (unable to resolve from 5133) with the loss of the 1288 bp fragment (*). The *Bam*HI fragment corresponding to the *vIL8* gene in 686BAC is 3092 bp (*), whereas in 686BAC-∆vIL8 it is 2414 bp (*). Digestion of 686BAC-∆Meq∆vIL8 with *Bam*HI resulted in 5404 bp and 2414 bp fragments, with the loss of 1288 bp (*) and 3092 bp fragments. On the other hand, the *Eco*RI fragment corresponding to the *meq* gene in 686BAC is 2456 bp (*), whereas in 686BAC-∆Meq it is 1439 bp (*). The *Eco*RI fragment corresponding to the *vIL8* gene in 686BAC is 9476 bp, which is reduced to 8798 bp in 686BAC-∆vIL8 (unable to resolve from 9476 bp fragment). Digestion of 686BAC-∆Meq∆vIL8 with *Eco*RI resulted in 8798 bp (unable to resolve from 9476 bp fragment) and 1439 bp fragments (*).

In addition, PCR amplification was performed to confirm the deletions of *meq* and/or *vIL8*, and the MDV *ribonucleotide reductase* (*RR)* gene served as an internal control, as it should not be affected by the mutation process. As shown in Figure 1C, the *RR* gene was amplified from genomic DNA isolated from chicken embryonic fibroblasts (CEF) infected with all viruses (Figure 1C, *RR*: lanes 1–5), while *meq* was amplified only from 686, 686BAC, and 686BAC-∆vIL8 viruses (Figure 1C, *meq*: lanes 1, 2, and 4), and *vIL8* was amplified only from 686, 686BAC, and 686BAC-∆Meq (Figure 1C, *vIL8*: lanes 1, 2, and 3). Neither *meq* nor *vIL8* were amplified from genomic DNA isolated from 686BAC-∆Meq∆vIL8 virus infected CEF (Figure 1C, *meq* and *vIL8*, lanes 5). The manipulated regions of *meq* and *vIL8* were sequenced using PCR products and no unexpected mutations were detected.

Immunofluorescence assay (IFA) of BAC transfected cells showed that expression of viral protein pp38 and plaque size were similar in parental and deletion mutant viruses infected CEF (Figure 1D). In addition, parental 686BAC, 686BAC-∆Meq, 686BAC-∆vIL8, and 686BAC-∆Meq∆vIL8 showed similar in vitro growth kinetics (Figure 1E), suggesting that deletion of *meq* and/or *vIL8* did not affect the growth of any of the viruses in cell culture.

### 3.2. In Vivo Replication and Reactivation of meq or vIL8 Single and Double Deletion Viruses

**Figure 2:**
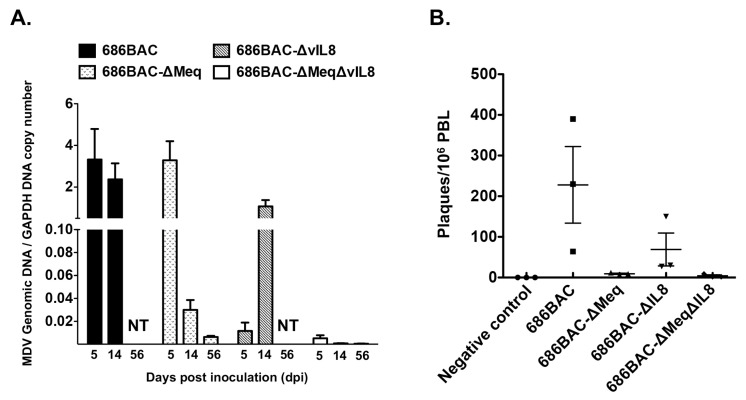
In vivo replication and reactivation of *meq* and *vIL8* single and double deletion mutant viruses. (**A**) Virus genome copy number in spleen of inoculated chickens. Genomic DNA were extracted from splenocytes of inoculated chickens at 5, 14, and 56 days post-inoculation (dpi) and virus genome copy number was measured by qPCR. 686BAC and 686BAC-ΔvIL8 viruses inoculated chickens were not tested (NT) at day 56, as all the chickens in those groups had died. Results are presented as average MDV genome copies per GAPDH copy of three chickens, and error bars represent standard error of the mean (SEM). (**B**) Virus reactivation assay. CEF were co-seeded with 10^6^ peripheral blood lymphocytes (PBL) isolated from 686BAC, 686BAC-ΔMeq, 686BAC-ΔvIL8, or 686BAC-ΔMeqΔvIL8 virus inoculated chickens at 14 dpi. Plaques were counted 7 days after infection. Data present average plaque numbers of three chickens ± SEM.

Early cytolytic infection of MDV occurs around 2–6 days post-infection followed by the latent phase, starting at 7–8 days post-infection, and transformed cells appear as early as 2 weeks post-infection. To examine the replication of deletion mutant viruses in vivo, one-day-old MDV Ab– chickens were inoculated with 2000 plaque-forming units (PFU) of parental 686BAC, 686BAC-∆Meq, 686BAC-∆vIL8 or 686BAC-∆Meq∆vIL8 viruses and their replication rates (virus genome copy number) were measured in spleen samples taken at 5, 14, and 56 days post-inoculation (dpi). At 5 dpi, parental 686BAC and 686BAC-∆Meq inoculated chickens had similar virus genome copy numbers and these were significantly higher than for 686BAC-∆vIL8 and 686BAC-∆Meq∆vIL8 inoculated chickens (Figure 2A). At 14 dpi, the genome copy number in 686BA-∆Meq and 686BAC-∆Meq∆vIL8 inoculated chickens was significantly lower than in the parental 686BAC group. While the genome copy number in the 686BAC-∆vIL8 group increased by ~100× compared to day 5, at 14 dpi it was still lower than in the parental 686BAC group (Figure 2A). These results are consistent with the virus reactivation assay results at 14 dpi (Figure 2B). At 56 dpi, the genome copy number in all groups was low or not tested (NT), due to the death of inoculated chickens (Figure 2A).

**Figure 3:**
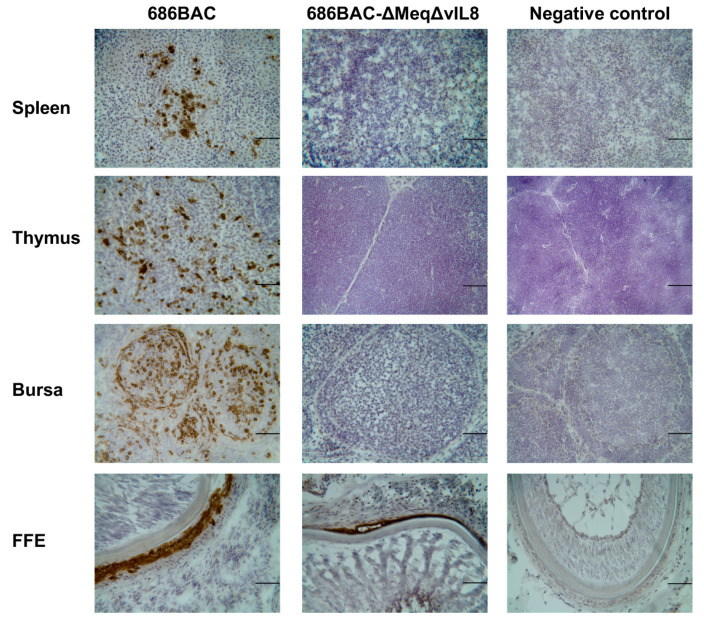
Replication of *meq* and *vIL8* double deletion virus in lymphoid organs and feather follicle epithelium (FFE) of inoculated chickens. At 5 or 14 days post-inoculation (dpi), lymphoid organs (spleen, thymus, and bursa) and feather follicle epithelium (FFE) were collected, respectively, from chickens inoculated with 686BAC or 686BAC-∆Meq∆vIL8 viruses, or negative control chickens. Tissue samples were frozen immediately using the O.C.T. (optimal cutting temperature) compound and 6 to 8 µm-thick cryostat sections were stained with MDV pp38 specific monoclonal antibody. Scale bar = 50 µm.

To further examine virus replication in chickens, MDV pp38 expression was evaluated by immunohistochemistry (IHC) assay. Three MDV Ab– chickens inoculated with parental 686BAC or 686BAC-∆Meq∆vIL8 viruses were euthanized at 5 dpi and their lymphoid organs (thymus, bursa of Fabricius, and spleen) examined for pp38 expression. Our results show that there were high levels of pp38 expression in the lymphoid organs of parental 686BAC inoculated chickens, while almost no pp38 expression was detected in 686BAC-∆Meq∆vIL8 inoculated chickens (Figure 3). Taken together, the in vivo MDV genome copy number data and pp38 expression results suggest that double deletion of *meq* and *vIL8* significantly reduces virus replication in chickens. 

We also examined the replication of 686BAC-∆Meq∆vIL8 virus in feather follicle epithelium (FFE), where fully infectious viral particles are produced. IHC results showed that chickens inoculated with 686BAC-∆Meq∆vIL8 virus had levels of pp38 expression similar to parental 686BAC virus at 14 dpi (Figure 3, FFE), suggesting that double deletion of *meq* and *vIL8* did not abrogate virus replication in FFE.

### 3.3. Evaluation of Lymphoid Organ Atrophy and Pathogenesis Induced by Inoculation of meq or vIL8 Single and Double Deletion Viruses

We have reported earlier that the deletion of *meq* results in the complete loss of oncogenicity but does not eliminate lymphoid organ atrophy; while the deletion of *vIL8* significantly reduces MDV transformation, due to reduced virus replication in lymphoid organs [21,33]. Therefore, we hypothesized that the deletion of both genes would result in a non-oncogenic virus that does not cause lymphoid organ atrophy. 

**Figure 4:**
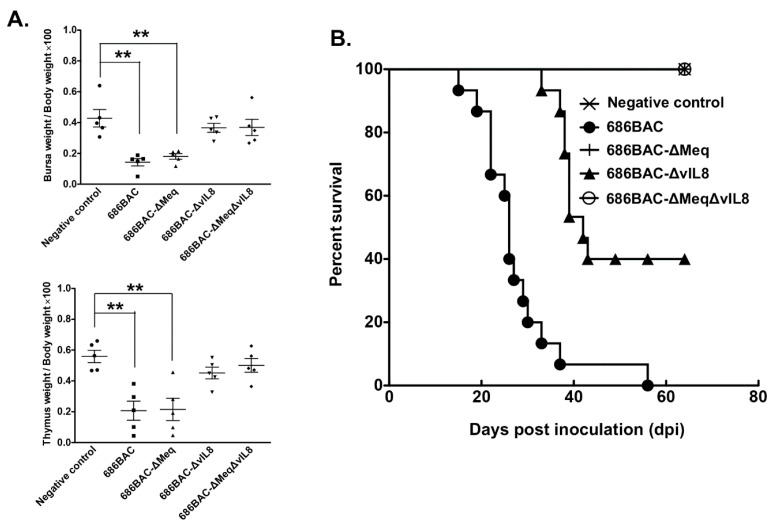
Pathogenesis studies of *meq* and *vIL8* single and double deletion mutant viruses. One-day-old SPF MDV Ab– chickens were inoculated with 2000 plaque-forming units (PFU) of 686BAC, 686BAC-ΔMeq, 686BAC-ΔIL8 or 686BAC-ΔMeqΔIL8 viruses, or kept uninoculated and served as negative control. Chickens were maintained in isolation for 65 days and daily mortality was recorded. (**A**) Lymphoid organ atrophy. At 14 days post-inoculation (dpi), five chickens from each group were euthanized, the lymphoid organs collected, and the relative bursa and thymus to body weight was determined. Results represent mean value with error bars representing standard error of the mean. **: *p* < 0.01. (**B**) Survival curves of chickens inoculated with the indicated viruses or uninoculated control group.

To evaluate the lymphoid organ atrophy and pathogenic properties of 686BAC-∆Meq∆vIL8 virus, one-day-old MDV Ab– chickens were inoculated with parental and mutant viruses or remained uninoculated and served as negative control. At 14 dpi, lymphoid organs (bursa and thymus) collected from five chickens from each group were weighted. As shown in Figure 4A, compared to negative control chickens, parental 686BAC and 686BAC-∆Meq viruses, but not 686BAC-∆vIL8 and 686BAC-∆Meq∆vIL8 viruses, induced severe bursa and thymus atrophy in inoculated chickens. 

To further evaluate the pathogenic properties of 686BAC-∆Meq∆vIL8, all inoculated and uninoculated chickens were closely monitored for 65 days. Our results show that MD associated mortality began at 16 dpi in the parental 686BAC group, and all chickens died before the end of the experiment, while MD associated mortality began at 37 dpi in 686BAC-∆vIL8 group, and 6 out of 15 (40%) chickens survived for the duration of the experiment (Figure 4B). All the chickens that died in the 686BAC and 686BAC-∆vIL8 groups had MD specific gross tumors. However, no MD specific mortality and tumors were observed in the uninoculated control, 686BAC-∆Meq, and 686BAC-∆Meq∆vIL8 groups (Figure 4B). Taken together, these results indicated that 686BAC-∆Meq∆vIL8 was fully attenuated. 

### 3.4. 686BAC-∆Meq∆vIL8 Provides Protection Comparable to CVI988 against Challenge with a vv+ MDV

**Figure 5:**
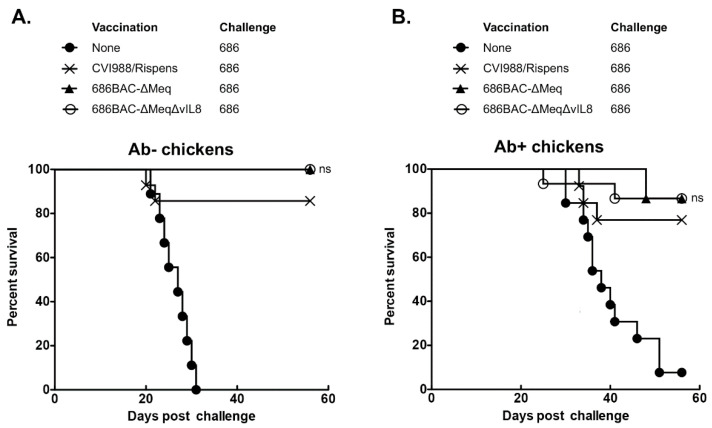
Protection studies of *meq* and *vIL8* double deletion virus in MDV maternal antibodies negative (Ab–) and positive (Ab+) chickens. Fifteen one-day-old SPF MDV Ab– (**A**) and commercial MDV Ab+ (**B**) chickens were unvaccinated or vaccinated with 2000 plaque-forming units (PFU) of 686BAC-∆Meq, 686BAC-∆Meq∆vIL8, or CVI988/Rispens subcutaneously. Five days later, vaccinated and unvaccinated control chickens were challenged subcutaneously with 500 PFU of MDV 686 strain virus. Survival curves of each group are presented. The trends of chicken survival over time were examined with LogRank and Wilcoxon tests. ns: no significant difference to CVI988/Rispens vaccinated group.

The protection efficacy of 686BAC-∆Meq and 686BAC-∆Meq∆vIL8 was compared to that of CVI988, the gold standard MD vaccine, in both Ab– and Ab+ chickens challenged with a vv+ MDV (686) strain. In the Ab– group, MD specific mortality and gross tumors were observed in 100% of the unvaccinated chickens (Figure 5A and Table 2). In the CVI988 vaccinated group, MD specific mortality was observed in 13% of the chickens, while no mortality was observed in the 686BAC-∆Meq or 686BAC-∆Meq∆vIL8 vaccinated groups (Figure 5A). On the other hand, the incidence of MD specific gross tumors was 20% in the CVI988 vaccinated group, while no apparent MD specific gross tumors were observed in the 686BAC-∆Meq and 686BAC-∆Meq∆vIL8 vaccinated groups (Table 2). The protective index (PI) values were 80%, 100%, and 100% for CVI988, 686BAC-∆Meq, and 686BAC-∆Meq∆vIL8, vaccinated groups, respectively (Table 2). Even though the PI values of groups vaccinated with 686BAC-∆Meq or 686BAC-∆Meq∆vIL8 were higher than the group vaccinated with CVI988, the differences were not significant (Table 2).

In Ab+ chickens, MD specific mortality in the unvaccinated group was 92%, while in the CVI988, 686BAC-∆Meq, and 686BAC-∆Meq∆vIL8 vaccinated groups it was 21%, 14%, and 13%, respectively (Figure 5B). On the other hand, MD specific gross tumors were apparent in 29%, 7%, and 13% of the CVI988, 686BAC-∆Meq, and 686BAC-∆Meq∆vIL8 vaccinated chickens, respectively (Table 2). The PI values observed were 71%, 93%, and 87% for the CVI988, 686BAC-∆Meq, and 686BAC-∆Meq∆vIL8 vaccinated groups, respectively (Table 2), which are not significantly different. Taken together, these results indicate that 686BAC-∆Meq∆vIL8 protection against vv+ MDV challenge is comparable to that of CVI988.

## 4. Discussion

Since the early 1970s, the poultry industry has relied on the use of vaccines to control losses due to MD. These vaccines, although effective at preventing mortality and tumor formation, do not prevent infection by field strains, and are thought to have contributed to the emergence of more virulent field strains [5,41,42,43]. The lack of more effective MD vaccines has led the poultry industry to rely on the use of multiple vaccine doses and/or multivalent vaccines [44], significantly increasing vaccination cost. Although several MDV-derived vaccine candidates have been generated, only few have been successful. MDV CVI988/Rispens is the most efficacious vaccine in the market and is considered the ‘gold-standard’ [45,46,47]. 

The mechanisms of MD vaccine protection are still not well understood, but it has been suggested that in order to be effective, an MD vaccine has to be able to replicate to sufficient levels to stimulate an immune response. Attempts have been made to attenuate highly virulent MDV strains, by serial passage in cell culture, to develop more efficacious MD vaccines. It has been shown that partially attenuated strains, which were still mildly oncogenic, induced higher protection than CVI988/Rispens, while fully attenuated passages conferred only limited protection [48,49]. It was also been shown that highly protective vaccines replicate to higher levels than low protective vaccines in lymphoid organs of vaccinated chickens [50]. These data suggest that the protection efficacy of MD vaccines is highly associated with its replication. 

A number of laboratories have used molecular techniques to identify and study MDV genes involved in MDV pathogenesis, viral replication, or tumor formation. These techniques have facilitated, through the deletion or mutation of genes associated with pathogenesis, the development of attenuated recombinant viruses which could be used as potential vaccines. One of the most promising candidates was an MDV mutant virus lacking both copies of the *meq* oncogene (MDV-ΔMeq) [21]. Deletion of *meq* rendered the virus non-oncogenic, while maintaining a normal early cytolytic infection, and proved to be an effective vaccine candidate in laboratory and/or field conditions [29,30]. However, like the parental vv MDV, MDV-ΔMeq causes lymphoid organ (bursa and thymus) atrophy in highly susceptible MDV Ab– chickens [31,32], an important safety concern that has interfered with its commercialization. In order to improve the safety of MDV-ΔMeq virus, Lee et al. attempted to further attenuate it by serial passage in cell culture. Although at passage 40 MDV-ΔMeq virus did not cause lymphoid organ atrophy in MDV susceptible chickens, the protection efficacy in MDV Ab+ chickens was reduced [32], potentially due to the introduction of random mutations in genes essential for virus replication. Thus, this approach was not satisfactory for improving the safety of MDV-ΔMeq virus without negatively affecting its efficacy as vaccine.

An alternative strategy, used in this study, was to introduce known secondary mutations in the MDV genome, by deleting or mutating a second gene involved in virus replication. It has been shown that the introduction of a point mutation in UL5, a helicase primase subunit involved in virus replication in vivo, provided partially protection against v and vv MDV [51]. A subsequent study showed that the introduction of the UL5 point mutation in MDV-ΔMeq virus eliminated the lymphoid organ atrophy associated with MDV-ΔMeq virus; however, the protection efficacy of the resulting virus (PI = 51%) was significantly lower than MDV-ΔMeq virus (PI = 94%) and CVI988/Rispens (PI = 94%) [52]. Thus, the introduction of a random or site-specific mutation in certain gene/s involved in virus replication cannot improve the safety of the MDV-ΔMeq virus without affecting its protection efficacy. 

It was previously shown that the deletion of *vIL8* from the MDV genome resulted in reduced oncogenicity [20,33], primarily due to severely restricted early cytolytic replication in lymphoid organs [33]. Interestingly, MDV-ΔvIL8 still conferred good protection against challenge with vv+ MDV strains [34]. On the basis of these characteristics, we hypothesized that the double deletion of *meq* and *vIL8* may reduce lymphoid organ atrophy but still retain the protective efficacy of MDV-ΔMeq. In the present study, we generated a double gene deletion mutant virus in which both *meq* and *vIL8* were deleted from the vv+ MDV strain 686 (686BAC-ΔMeqΔvIL8). Like the single deletion mutant viruses, 686BAC-ΔMeqΔvIL8 replicated well in vitro (Figure 1). Further characterization of this virus in vivo showed that it replicates to a significantly lower level than parental 686BAC and 686BAC-ΔMeq viruses in spleen, as measured by qPCR at 5, 14, and 56 dpi (Figure 2A). Similarly, during early cytolytic infection (day 5), 686BAC-ΔMeqΔvIL8 showed very limited to no viral antigen expression in lymphoid organs (spleen, thymus, and bursa) (Figure 3). These results suggest that the double deletion of *meq* and *vIL8* did not affect the virus replication in vitro or FFE, but significantly reduced its replication in lymphoid organs (spleen, thymus, and bursa). Furthermore, 686BAC-ΔMeqΔvIL8′s limited replication during the early cytolytic phase was comparable to that of 686BAC-ΔvIL8 (Figure 2A). This limited virus replication in the lymphoid organs probably contributed to the reduction of lymphoid organ (bursa and thymus) atrophy (Figure 4A) and mortality rate (Figure 4B), in Ab– chickens inoculated with 686BAC-ΔMeqΔvIL8. 

Additionally, in agreement with previous results, 686BAC-ΔvIL8 replicated poorly during early cytolytic infection (day 5) resulting in a low viral load compared to parental 686BAC and 686BAC-ΔMeq viruses (Figure 2A). However, since the 686BAC-ΔvIL8 virus causes lymphocyte transformation, by 14 dpi there was a ~100 fold increase in virus genome copy number. On the other hand, at the same time point, the genome copy number in the 686BAC-ΔMeq group decreased by ~100 fold in agreement with the lack of transformation. As a result of the role of Meq and vIL8 in virus replication and transformation, the virus genome copy number of 686BAC-ΔMeqΔvIL8 virus was low during early cytolytic infection (due to the absence of vIL8) and absent during the transformation phase (due to the absence of Meq). Interestingly, we and others have earlier shown that MDV-ΔMeq is able to confer long term protection even though the virus could not be efficiently recovered from infected chickens after the initial cytolytic phase [29,53]. Similarly, in this study, we showed that 686BAC-ΔMeqΔvIL8 replicated to low levels during the early cytolytic infection (due to *vIL8* deletion) and was defective in latency and/or reactivation and transformation (due to *meq* deletion) (Figure 2), while it was still capable of inducing a vigorous immune response able to provide excellent protection against challenge with a vv+ MDV (Figure 5 and Table 2). These results suggest that the 686BAC-ΔMeqΔvIL8 may use an alternative mechanism of protection, which needs further investigation. We speculate that even though the replication of 686BAC-ΔMeqΔvIL8 is severely impaired in lymphocytes, its replication in other cells, yet to be identified, may be important for MD protection. In addition, a single trial of the animal experiment was performed in both MDV maternal Ab– and Ab+ chickens, future studies will include a larger number of chickens to verify the data and study the potential mechanisms behind the protection conferred by the double deletion mutant virus.

Another drawback of currently available MD vaccines, including the “gold standard”, CVI988/Rispens, is that they cannot completely overcome field virus infection-induced immunosuppression (MDV-IS). It has been recently shown that infection of highly virulent MDV could induce MDV-IS in chickens, which is classified into three phases, including early-MDV-IS (early IS associated with early cytoytic infection of MDV in lymphoid organs), late-MDV-IS-R (late IS associated with reactivation of MDV), and late-MDV-IS-T (late IS associated with development of tumors) [54]. Interestingly, early-MDV-IS and late-MDV-IS-T are well controlled by current MD vaccines; however, only MDV-ΔMeq has been shown to protect against late-MDV-IS-R [55]. Future studies will examine if 686BAC-ΔMeqΔvIL8 is capable of overcoming MDV-IS, especially late-MDV-IS-R.

MDV undergoes secondary replication in the FFE and sheds infectious virus into the environment through dander, infecting susceptible chickens [3]. We showed earlier that deletion of either *vIL8* [33] or *meq* [21] genes did not interfere with the ability of the virus to replicate in the FFE, suggesting that these genes are not essential for horizontal transmission. Here, we showed that the deletion of both *vIL8* and *meq* did not affect virus replication in the FFE (Figure 3). Thus, it is our expectation that the 686BAC-ΔMeqΔvIL8 mutant virus may spread efficiently among chickens even though it was unable to replicate effectively and transform lymphocytes in chickens. Since the double deletion virus reported here replicated efficiently in the FFE (Figure 3), it would be interesting to study if it could interfere with the transmission of field viruses, thus slowing the progression of evolution of field viruses to greater virulence. 

## 5. Conclusions

In summary, in this report, we show that the double deletion of *meq* and *vIL8* did not affect virus growth in cell culture and FFE but significantly impaired virus replication in lymphoid organs. In addition, we demonstrate that the 686BAC-ΔMeqΔvIL8 virus overcame the disadvantage of 686BAC-ΔMeq virus-induced lymphoid organ atrophy while providing good protection against vv+ MDV challenge. Our study supports double gene deletion/mutation as a new strategy that can be exploited to generate the next generation of MD vaccines.

## Figures and Tables

**Table 1:** Primers used in this study.

Primers	Sequences (5′ to 3′)	Purposes
vIL8Kan-F	**aaaatcggaaaaaaaagtgccttcttttaattacaggaggtagcaattaa** aggatgacgacgataagtaggg	Amplification of Kan^R^ cassette with MDV sequences flanking *vIL8* gene
vIL8Kan-R	**gatatataatgcagggggtgtgggtttgatgagcagttggggcggcaaaattaattgctacctcctgtaattaaaagaaggcactttttttttccgatttt** caaccaattaaccaattctgattag	
MeqKan-F	**cttgcaggtgtataccagggagaaggcgggcacggtacaggtgtaaagag** aggatgacgacgataagtaggg	Amplification of Kan^R^ cassette with MDV sequences flanking *meq* gene
MeqKan-R	**aacatggggcatagacgatgtgctgctgagagtcacaatgcggatcatcactctttacacctgtaccgtgcccgccttctccctggtatacacctgcaag** caaccaattaaccaattctgattag	
vIL8-F	gccaagcttcgaggagtcaaaatcgg	Amplification of *vIL8* gene
vIL8-R	gccgaattcggtggagacccaataac	
Meq-F	ccgcacactgattcctag	Amplification of *meq* gene
Meq-R	ccttatgttgatcttccca	
RR-F	ccgcgatcgttaggttgggtatta	Amplification of *RR* gene
RR-R	ccttatgttgatcttccca	
ICP4-F	ttattgccccgtactcaccg	Viral genomic copy number detection
ICP4-R	catttaaagtctttccatgccaaac	
GAPDH-F	gtcaacggatttggccgtat	Viral genomic copy number detection
GAPDH-R	ccacttggactttgccagaga	

F: forward; R: reverse; Underlined sequences are from pEPkan-S plasmid used to amplify the *Kan*^R^ gene cassette; Sequences in bold indicate MDV genome sequences flanking *vIL8* or *meq* genes.

**Table 2:** Protective efficacy of CVI988/Rispens, 686BAC-∆Meq, and 686BAC-∆Meq∆vIL8 against challenge with vv+ MDV 686 strain in MDV Ab– and Ab+ chickens.

Vaccination	Challenge	Ab–		Ab+	
		Tumors (%) ^1^	PI	Tumors (%)	PI ^3^
CVI988/Rispens	686	3/15 (20)	80 ^a^	4/14 (29)	71 ^a^
686BAC-∆Meq	686	0/13 (0)	100 ^a^	1/14 (7)	93 ^a^
686BAC-∆Meq∆vIL8	686	0/13 (0)	100 ^a^	2/15 (13)	87 ^a^
None	686	12/12 (100)	NA ^2^	13/13 (100)	NA
None	None	0/10 (0)	NA	0/15 (0)	NA

**^1^** Tumors (%) = Incidence of Marek’s disease specific gross tumors; **^2^** NA = Not applicable; **^3^** PI = Protective index. Indices with different letter superscripts are statistically different (*p* < 0.05).

## Data Availability

The data presented in this study are available on request from the corresponding author.
